# Exploratory Assessment of Nutritional Evaluation Tools as Predictors of Complications and Sarcopenia in Patients with Colorectal Cancer

**DOI:** 10.3390/cancers15030847

**Published:** 2023-01-30

**Authors:** Isabel M. Vegas-Aguilar, Patricia Guirado-Peláez, Rocío Fernández-Jiménez, Hatim Boughanem, Francisco J. Tinahones, Jose Manuel Garcia-Almeida

**Affiliations:** 1 Department of Endocrinology and Nutrition, Virgen de la Victoria University Hospital, 29010 Málaga, Spain; 2 Institute of Biomedical Research in Malaga (IBIMA)—Bionand Platform, University of Malaga, 29590 Málaga, Spain; 3 Department of Endocrinology and Nutrition, Quironsalud Málaga Hospital Av. Imperio Argentina, 29004 Málaga, Spain; 4 Spanish Biomedical Research Center in Physiopathology of Obesity and Nutrition (CIBERObn), Instituto de Salud Carlos III, 28029 Madrid, Spain; 5 Department of Medicine and Dermatology, Faculty of Medicine, University of Malaga, 29010 Málaga, Spain

**Keywords:** phase angle, muscle mass, muscle quality, complications, sarcopenia, colorectal cancer

## Abstract

**Simple Summary:**

Patients with colorectal cancer (CRC) are largely malnourished, which decreases overall survival. In this study, we found that phase angle in patients with CRC, measured by BIA, had a good diagnostic accuracy for detecting cancer complications in females and the risk of sarcopenia in males. These sex bias differences are relevant to understanding the nutritional status of CRC patients and their personalized nutritional treatment.

**Abstract:**

Background: Patients with colorectal cancer (CRC) are largely malnourished, which decreases overall survival and treatment efficacy and increases mortality rates. We hypothesize that angle phase might be associated with the risk of sarcopenia as well as cancer complications in patients with CRC. The inclusion of various nutritional status indicators and clinical cancer outcomes can result in significant variability. Therefore, the objective of this study was to perform an exploratory analysis of nutritional evaluation tools used to assess body composition and muscle quality in patients with CRC, in order to predict cancer complications and survival rate. Methods: A total of 127 patients with CRC were included in this study. Bioelectrical impedance analysis and body composition were performed, which we used to obtain phase angle (PhA) values. Muscle function was assessed by hand-grip strength (HGS) and muscle quality and adipose tissue depot were performed using ultrasound techniques. Results: This study showed that there were significant differences in body composition between females and males, as well as in muscle quantity and quality. PhA was highly correlated with quadriceps rectus femoris of cross-sectional area (RF-CSA), circumference of quadriceps rectus femoris (RF-CIR), superficial subcutaneous abdominal fat (S-SAT), as well as HGS (*p* < 0.05). PhA was also correlated with water content in females, and with muscle mass and quality in males (*p* < 0.05). Specifically, we found that PhA was a good predictor for cancer complications in women and the risk of sarcopenia in men. In the linear model controlled for age and body mass index (BMI), high PhA value was associated with a decreased risk of complications in females (Odds Ratio (OR) = 0.15, 95% CI: 0.03–0.81, *p* < 0.05). High PhA value was associated with a decreased risk of sarcopenia in males (OR = 0.42, 95% CI: 0.19–0.95, *p* < 0.05). In addition, Receiving Operating Characteristics (ROC) curve analysis showed that PhA had a good diagnostic accuracy for detecting cancer complications in females (Area under curve (AUC) = 0.894, 95% CI: 0.88–0.89, *p* < 0.05) and the risk of sarcopenia in males (AUC = 0.959, 95% CI: 0.91–0.92, *p* < 0.05). Conclusions: PhA can accurately predict oncological complications in women and sarcopenia in men. These differences are relevant to understanding the nutritional status of patients with CRC and their personalized nutritional treatment.

## 1. Introduction

Colorectal cancer (CRC)—a malignant neoplasm of the colon, rectum, and appendix—has already become a major health problem, being the third most commonly diagnosed malignancy and the second leading cause of cancer deaths in the world [1]. Great efforts are currently being made to decrease mortality rates in patients with CRC, including the use of effective screening tools to detect CRC early as well as to improve quality of life by diminishing cancer-related complications [2]. Unfortunately, the majority of patients with CRC suffer from side effects, which can lead to a reduction in energy intake and serious nutritional problems, such as loss of body weight and, specifically, loss of muscle mass, which may decrease overall survival [3,4]. In addition, most patients with CRC who undergo chemo- and radiotherapy develop malnutrition, sarcopenia, and/or cachexia [5], in which the rate of malnutrition and muscle wasting are independent of body mass index (BMI), and, at the same time, are negatively associated with overall survival and mortality [6]. Traditionally, BMI and anthropometric measurements are generally believed to predict malnutrition and sarcopenia. However, these phenotypes may be obscured by water retention and changes in the ratio of muscle mass to fat accumulation, particularly in cancer patients [7]. As a result, body composition can only provide information about nutritional deficits, whereas detailed body composition assessment can reveal real changes in metabolism, including muscle quality and fat depot [8].

Currently, phase angle (PhA) from bioelectrical impedance analysis (BIA) is a powerful tool available to measure cell mass and cellular damage, providing advice regarding not only body composition, but also nutritional status and overall mortality in patients with different pathologies [9,10]. In the cancer context, PhA has been widely used to assess the nutritional status of those patients that suffer from nutritional complications. For instance, Pereira et al. (2018) conducted a systematic review of nine studies and 1496 patients with cancer and found a significant association between PhA and overall survival [11], which was further confirmed by another systematic review conducted by Arab et al. (2021) [12]. In addition, a study conducted by Wei et al. (2021) found that PhA was correlated with sarcopenia in 445 patients with cancer, with an area under curve (AUC) of 0.785 predicting the condition [13], which was further observed in different studies [14,15]. However, data about PhA and the risk of complications are scarce, despite the fact that complications in cancer have been related to muscle quality [16]. As mentioned, several studies have assessed the utility of PhA in determining nutritional status, emphasizing its predictive value for cancer outcomes. However, more studies are needed to deepen the role of PhA in predicting quality of muscle in patients with CRC. Predicting sarcopenia and complications in cancer patients may improve overall survival in cancer patients and decrease mortality rates caused by malnutrition and muscle wasting.

Thus, in the present study, we hypothesize that angle phase might be associated with the risk of sarcopenia and cancer complications in patients with CRC. The inclusion of various nutritional status indicators and clinical cancer outcomes can result in significant variability. Our objective, therefore, was to conduct an exploratory analysis of different methods used to assess body composition and muscle quality in CRC patients. Further, we focused in our study on the potential of PhA as a predictive marker for cancer complications and sarcopenia, as these outcomes are risk factors for mortality. Appropriate identification of predisposition in patients with CRC using PhA might help to introduce specific nutritional interventions to prevent complications and sarcopenia and increase overall survival of cancer patients.

## 2. Materials and Methods

### 2.1. Study Design

This cross-sectional study included 127 patients recruited from University Hospital “Virgen de la Victoria” (Málaga, Spain) from October 2019 to June 2020. All patients with CRC were diagnosed by medical specialists, using diagnostic tools such as colonoscopy followed by biopsy, and their medical records/pathological examinations were complete. All biopsy samples were classified according to histology for pathologists features, and the World Health Organization Classification of Tumours of the Digestive System (2016) [17]. Patients were typically treated by chemotherapy and radiotherapy. For the radiotherapy, patients with CRC received radiation therapy with a total dose of 50 Gy delivered in 25 fractions of 2 Gy/fraction. For the first chemotherapy line, patients received fluoropyrimidine. Complications included paralytic ileus, bladder injury, fever, pancreatic fistula, septic shock, dehiscence, acute edema, hemorrhage, colon ischemia, or abscess. Treatment modality included surgery of different types (low anterior resection, sigmoidectomy, descending colon resection, left and right hemicolectomy, colostomy, or proctectomy. All patients were included in a pre-habilitation protocol with immunonutrition (Atempero^®^ Vegenat healthcare, S.L., Spain) and a physical activity plan. All patients provided written informed consent. The study was reviewed and approved by the Ethics Committee of University Hospital Virgen de la Victoria, Málaga, Spain (1649-N-22).

### 2.2. Body Composition

Body-composition analysis was obtained using Nutrilab (Akern, Florence, Italy). Impedance data derived using tetrapolar electrodes positioned on the right hand and foot are shown directly on an LCD touchscreen and stored in an internal memory. The CV% was evaluated in this cohort: the mean coefficients of variation for both parameters were []% intra-patient and []% inter-operator. Serial number > 2019 245 µA current at 50 kHz (±1%), Accuracy: Rz: ±0.1 Ω Xc: ±0.1 Ω, CV% < 1%. Capacitive resistance and resistance were obtained as described previously [10]. This measure provides a product in degrees as PhA and standardized phase angle (SPA), which comprises PhA adjusted by age and sex. Additional variables were also obtained from BIA, for example bioimpedance-derived parameters such as hydration (fluids percentage within the FFM values) and nutrition status (creatine excretion rate in mg/kg/24 h obtained from BCM values). All bioimpedance measurements were obtained from patients in a supine position on a bed. Data from BIA were categorized as fat-free mass (FFM) (kg) and FFM index (FFMI) (%); fat mass (FM) (kg) and FM index (FMI) (%); muscle mass (MM) (kg); skeletal muscle mass (SMM) (kg) and SM index (SMI) (%); intracellular water (ICW); total body water (TBW) (kg); and body cell mass (BCM) (kg). Height measurements were measured using a 2 mm sensitivity laser height rod. The dietitian collected all data at the start and end of the study. 

### 2.3. Nutritional Status and Muscle Quality

To measure muscle quality, we included handgrip strength, which was determined by means of the JAMAR-Dynamometer (J A Preston Corporation, New York, NY, USA). Both hands were tested, and three measurements were obtained. The final result from these measurements was reported as an average. Moreover, we used muscle and adipose tissue ultrasound techniques to measure the quality of muscle by testing the rectus femoris muscle of the quadriceps and subcutaneous and visceral adipose tissues; we used a HITACHI ALOKA F37 ultrasound scanner with an Aloka UST-5413 Linear Array transducer with a frequency range of 5.0–10.0 MHz in B-mode in transverse position (Hitachi Europe, Ltd., Tokyo, Japan). These techniques yield RF-CSA (rectus femoris of cross-sectional area), RF-CIR (circumference of the quadriceps rectus femoris), L-SAT (subcutaneous fat of leg), T-SAT (total subcutaneous abdominal fat), S-SAT (superficial subcutaneous abdominal fat), and VAT (preperitoneal or visceral fat) data. Finally, we also tested additional biochemical variables, such as prealbumin (mg/dL) (Atellica Siemens), albumin (g/dL), C reactive protein (CRP, mg/L) (Dimension EXL 200 Siemens), 25-hydroxyvitamin D (ng/mL), and the CRP/prealbumin ratio. Malnutrition was calculated using the following equation: the presence of malnutrition was detected using GLIM criteria with an FFMI lower than 17 for males and 15 for females [18]. Sarcopenia was assessed by the following formula: the presence of sarcopenia was indicated by an ASMMI lower than 7 for males and 5.5 for females, in accordance with the European Working Group on Sarcopenia in Older People 2 (EWGSOP2) [19].

### 2.4. Statistical Analysis 

The results are presented as mean ± standard deviation (SD) for continuous variables and as a number (percentage) for categorical variables. An exploratory analysis was conducted. A Student’s t-test or Wilcoxon test was performed according to the normality of the variables included in this study. A Pearson correlation coefficient test was performed. Linear and logistic regression analyses were also included in this study. The odds ratio (OR) (95% confidence intervals (CIs)) was obtained by logistic regression analysis. Principal component analysis was obtained from Xc (reactance) and Xz (resistance). Evaluation of the predictive property of PhA was based on the receiver operating characteristic (ROC) curve and AUC. Analyses and graphic representation were produced using R v.3.5.1 software (Integrated Development for R. RStudio, PBC, Boston, MA, USA), and the significance *p* value was set at *p* < 0.05.

## 3. Results

### 3.1. General Characterization of the Population Study

As summarized in Table 1, we divided our population by sex, including females and males as individual groups, due to their muscle difference. We did not observe a significant difference in age and BMI, nor in additional total cholesterol. However, differences mainly resided in those parameters related to the amount of fat and muscle between men and women (such as FM, FMI, or MM), as well as in the measurement tools used to measure muscle quality (PhA, SPA, RF-CSA, RF-CIR, T-SAT, or HG). Regarding clinicopathological variables, complications were more frequent in women than in men. However, no significant differences were observed between location, ileostomy/colostomy, or survival rates. The complete information on additional anthropometric variables is summarized in Supplementary Table S1.

### 3.2. Correlation Analysis between Nutritional Assessment Methods and Nutritional Status

**Figure 1:**
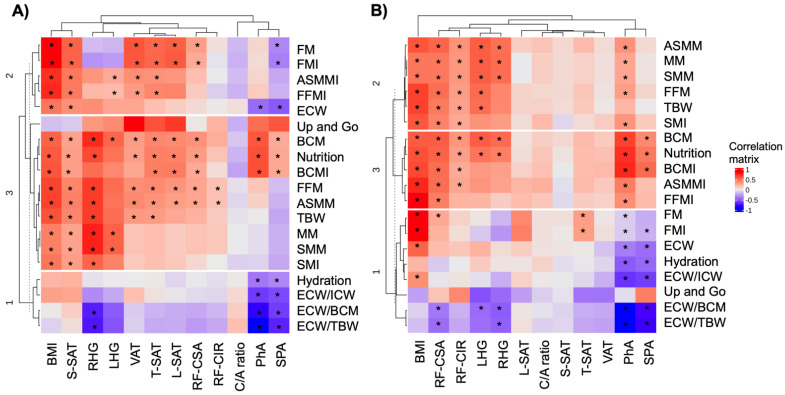
Correlation plots are presented to show association between body composition (*X*-axis) and body measurements (*Y*-axis) of (**A**) females and (**B**) males. A Pearson correlation coefficient test was conducted and the asterisk indicates significant correlation between variables according to the test (* *p* < 0.05). Abbreviations: BMI: body mass index; BIA: bioelectrical impedance analysis; CRP: C reactive protein; C/A: CRP/albumin; FM: fat mass; HGS: hand-grip strength; PhA: phase angle; RF-CIR: circumference of quadriceps rectus femoris; RF-CSA: rectus femoris cross-sectional area; SAT: subcutaneous adipose fat of leg (L), superficial abdominal (S) and total abdominal (T); SMI: skeletal muscle index; SPA: standardized phase angle.

To understand the relationship between nutritional evaluation tools and body composition and muscle quality in patients with CRC, an exploratory analysis was conducted. When we stratified by sex, and specifically focused on the study of females, we observed that most BIA and ultrasound measurements were good predictors of nutritional status and muscle quality (Supplementary Figure S1B contains the data from all participants). We found that PhA and SPA were associated with nutritional and hydration status. In addition, BMI, S-SAT, HG, CF-CSA, and RF-CIR were associated with muscle composition and muscle quality (Figure 1A). Furthermore, when we focused on the study of males, we found that PhA and SPA were associated with nutritional and hydration status, as well as muscle composition. The remaining measurement methods, such as BMI, CF-CSA, CF-CIR, and HG were correlated with nutritional status as well as muscle composition (Figure 1B). As a result, we assessed the relationship between PhA and other tools measuring muscle quality. We then found that PhA was correlated with most tools assessing the quality of muscle (Supplementary Figure S1A).

### 3.3. Correlation between Nutritional Assessment Methods and Cancer Outcomes

We looked at various nutritional status measurement tools to see how they correlated with major clinical and pathological cancer outcomes in CRC patients. To do this, we performed a point-biserial correlation analysis (Supplementary Figure S2A). When we specifically studied females, we observed that PhA was correlated with colorectal cancer complications and sarcopenia (Supplementary Figure S2B). In addition, C/A ratio was associated with cancer complications and hospital stays, whereas BMI and T-SAT were correlated with malnutrition and sarcopenia. When we specifically analyzed males, we observed a correlation between sarcopenia and PhA (Supplementary Figure S2C). Furthermore, LHG was associated with cancer complications, whereas VAT was correlated with hospital stays. Given that PhA was correlated with cancer complications and sarcopenia, we decided to focus on the study of PhA in relation to these variables, taking into account the difference in sex. As shown in Table 2, PhA was a good predictor of complications only in women, with a higher PhA value being associated with a decreased risk of cancer complications (showing an OR of 0.15 (CI 95% 0.03–0.81)). In the case of males, a higher LHG value was correlated with a decreased risk of complications in men (OR = 0.87 (CI 95% 0.76–1.00). PhA was a good predictor of sarcopenia. A higher PhA value was associated with a decreased risk of sarcopenia (OR of 0.42 (CI 95% 0.19–0.95)), in addition to a higher BMI (OR = 0.42 (CI 95% 0.24–0.74)) and a higher RF-CSA (OR = 0.51 (CI 95% 0.26–0.97)). After controlling for confounding variables, such as age and BMI, the regression analysis showed that PhA remained associated with complications and sarcopenia in women, yielding an HR of −2.52 (1.19) and −5.26 (278), respectively. As for males, PhA and SPA showed an HR of −2.29 (0.96) and −1.04 (0.50), respectively (Table 3).

### 3.4. PhA as a Prognostic Predictor of Complications and Sarcopenia in Colorectal Cancer

**Figure 2:**
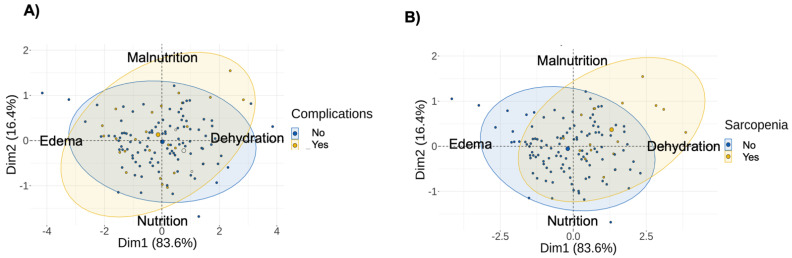
A principal component analysis using reactance and resistance as variables for the comparison between patients with complications and those without complications (**A**), and between patients with sarcopenia and those without sarcopenia (**B**).

Further, we studied the ability of PhA to determine the risk of complications in cancer or sarcopenia, since these variables are related to the risk of mortality. First, we conducted a PCA analysis using reactance and resistance as variables for the PCA. Figure 2A shows a not-well-defined cluster according to the complication’s variable. However, this graph generally predicts that these patients are malnourished, obese, and dehydrated, that is, on the three upper right and left axes. In Figure 2B, we observed that the two groups clearly cluster according to the sarcopenia variable. This graph generally predicts that these patients are malnourished and dehydrated, placing them in the upper right quadrant. On the other hand, we observed that PhA is a good predictor of sarcopenia in men. The predictive value of PhA on complications in women has an AUC value of 0.893, with a sensitivity of 80% and a specificity of 88.9% (*p* < 0.001). Meanwhile, in men, PhA has a predictive value with an AUC value of 0.959, a sensitivity of 90.9%, and a specificity of 92.3% (*p* = 0.026) (Table 4). SPA has a predictive value with an AUC value of 0.958, a sensitivity of 90.9%, and a specificity of 92.3% (*p* = 0.036).

## 4. Discussion

Nutritional status in patients with cancer is a crucial outcome, having a high bearing on the incidence of survival rates and postoperative complications. Although malnutrition is a well-indicated risk factor for cancer complications, the level of detection of malnutrition in patients with cancer is overestimated, and new tools are needed to best know the specific nutritional needs of cancer patients. Therefore, evaluation of the nutritional status of patients with CRC should be implemented as a routine clinical practice [20]. In this article, our exploratory analysis revealed that PhA is strongly associated with muscle quality, nutrition, and hydration, which means that using PhA in assessing nutritional needs for CRC patients is a useful tool in cancer management. Furthermore, we found that PhA is a good predictor for complications in females and sarcopenia in males, suggesting a sex difference in nutritional needs that must be considered for nutritional strategies in patients with CRC. To assess nutritional status and muscle mass in patients with CRC, we urgently need to take into account important differences between males and females that have been revealed in nutritional studies. 

As observed in Table 1, there were significant differences in the majority of indicators, such as water content, muscle mass quality, or fat mass, indicating that sex bias must be taken into account in the management of nutritional assessment of cancer patients. Indeed, Yoon et al. observed differences in body composition between male and female patients with CRC, indicating that these markers could be suitable as surrogate markers for sex-specific changes in cell composition and health status [21]. Overall, PhA has been considered a good predictor for cancer survival. Accordingly, Gupta et al. concluded that patients with CRC with a phase angle greater than 5.57 had an increased median survival than those with a PhA equal to or lower than 5.57 [22]. This is probably because well-nourished patients with CRC have a higher PhA score, indicating that PhA is a potential nutritional indicator in CRC [23]. However, Mauricio et al. did not find any association between SPA and postoperative complications, although low muscle mass was an independent risk factor for complications after controlling for confounding factors [24]. This suggests that additional studies are needed to clarify controversial results. In our study, we found that PhA was associated with complications in CRC only in females, indicating a sex-specific marker for cancer complications. After controlling for confounding variables, such as age and BMI, this association remained significant in the linear regression model, indicating a strong association. The AUC yielded a value of 0.893 for a cut-off of PhA of 4.85 (*p* = 0.001), suggesting that PhA has a good prognostic value for complications in females. However, further studies with a larger sample size are needed to specifically define the cut-off PhA value in predicting complications in cancer.

Sarcopenia is another cancer outcome that is of interest in nutritional assessment. Sarcopenia has been widely observed in patients with CRC, as a result of malnutrition and the presence of low muscle quantity and quality. Indeed, sarcopenia has been associated with a lower survival rate, an increased perioperative mortality, and a lower response to chemotherapy treatment, suggesting an urgent need for management of nutritional intervention [25]. 

In our study, sarcopenia was assessed using the formula described above in accordance with EWGSOP2 criteria. These criteria can vary according to the characteristics of patients. In our case, we included patients with CRC. While using EWGSOP2 is considered a good way to define sarcopenia in the cancer context, there is still no consensus on this; it may nevertheless be a good alternative for sarcopenia detection due to the ease with which it provides results [26]. As for PhA, sarcopenia was associated with PhA and other variables of muscle mass in univariate and multivariate regression analyses, conducted by Souza et al., indicating that PhA was an independent predictor of sarcopenia in patients with CRC [27]. Furthermore, Souza et al. found that PhA was highly correlated with muscle quality such as SMI and moderately correlated with HGS, maintaining these associations after controlling for age, sex, and body mass index, and with an optimal prognostic value using ROC curve analysis—indicating that PhA was a predictor of muscle abnormalities and function in patients with CRC [14]. As we found in our study, PhA was associated with the majority of measurement techniques for variables of muscle quality, such as CF-CSA, S-SAT, and HGS. Additionally, PhA was also associated with other muscle mass indicators, such as MM or SMI, but only in males. Indeed, PhA was associated with sarcopenia in CRC in males, but not in females, indicating a sex-specific marker for cancer complications. After controlling for confounding variables, such as age and BMI, this association remained significant in the linear regression model for PhA and SPA, indicating a strong association. The AUC yielded a value of 0.959 for a cut-off PhA value of 5.99 (*p* = 0.026), suggesting that PhA is of good prognostic value for sarcopenia in males.

There are various limitations to our study that should be mentioned. The cross-sectional design of this study may be one of these limitations. Furthermore, the participants were all from the same geographic area. They may not reflect the general population, since we cannot establish causal relationships between factors. The limited size of this study is another limitation. Although the sample size appeared to be modest, our recruited model was cross-sectional in design and had tight inclusion and exclusion criteria, which limited the number of participants. Finally, the influence of other variables may change nutritional status. Some factors, such as genetics, microbiota, aging, or physical activity were not taken into account in this study. To restrict the influence of these confounding factors, the authors modified the models by those controlling variables, such as age and BMI.

## 5. Conclusions

In summary, we found that PhA can accurately predict oncological complications in females and sarcopenia in males. This is relevant to treating nutritional status in cancer patients. Sex must be taken into account when personalizing the indicated nutritional treatment, with PhA being a good tool to improve the quality of life of cancer patients.

## Figures and Tables

**Table 1:** Summary descriptive table grouped by sex.

	All	Female	Male	*p* Value
	*N* = 127	*N* = 51	*N* = 76	
Demographic variables Age (years)	66.5 (8.89)	67.9 (7.77)	65.5 (9.51)	0.131
Weight (kg)	76.5 (15.8)	70.8 (15.2)	80.3 (15.2)	0.001 *
BMI (kg/m^2^)	27.6 (4.51)	27.6 (4.95)	27.5 (4.22)	0.889
BIA				
PhA (°)	5.79 (0.84)	5.39 (0.66)	6.07 (0.84)	<0.001 *
SPA (°)	0.76 (1.11)	1.06 (1.21)	0.56 (0.99)	0.016 *
FM (kg)	19.1 (7.40)	21.4 (7.34)	17.7 (7.14)	0.009
SMI (cm^2^/m^2^)	9.40 (1.82)	7.81 (1.23)	10.4 (1.32)	<0.001 *
Echography exploration				
RF-CSA	4.10 (1.61)	3.41 (1.58)	4.53 (1.48)	<0.001 *
RF-CIR	8.82 (1.53)	8.21 (1.62)	9.22 (1.34)	0.001 *
RF-Y axis (thickness)	1.29 (0.39)	1.12 (0.36)	1.40 (0.37)	<0.001 *
S-SAT	0.89 (0.55)	1.29 (0.58)	0.64 (0.33)	<0.001 *
T-SAT	1.66 (0.79)	2.08 (0.80)	1.39 (0.66)	<0.001 *
L-SAT	0.89 (0.99)	1.10 (0.57)	0.76 (1.17)	0.048 *
VAT	0.57 (0.39)	0.61 (0.43)	0.54 (0.37)	0.438
Functional measurement				
HGS (right hand)	27.6 (10.2)	20.5 (5.62)	31.5 (10.1)	<0.001
HGS (left hand)	26.3 (10.6)	18.3 (5.60)	30.6 (10.2)	<0.001 *
Biochemical variables				
Glucose (mg/dL)	104 (21.9)	105 (21.6)	104 (22.3)	0.808
Triglycerides (mg/dL)	127 (40.6)	127 (39.4)	127 (41.9)	0.941
Total cholesterol (mg/dL)	188 (40.7)	197 (41.3)	181 (39.3)	0.047 *
Albumin (g/dL)	3.66 (0.51)	3.67 (0.43)	3.65 (0.56)	0.861
CRP (mg/L)	8.73 (9.10)	7.26 (8.57)	9.88 (9.44)	0.213
25-hydroxyvitamin D (ng/mL)	20.2 (7.61)	18.3 (7.89)	22.4 (6.85)	0.081
Clinicopathological variables TNMs:				0.553
T1 + T2	35 (29.7%)	12 (25.5%)	23 (32.4%)	
T3 + T4	83 (70.3%)	35 (74.5%)	48 (67.6%)	
Cancer location:				1.000
Colon	80 (64.0%)	32 (64.0%)	48 (64.0%)	
Rectum	45 (36.0%)	18 (36.0%)	27 (36.0%)	
Treatment ^#^:				0.733
No	46 (36.8%)	17 (34.0%)	29 (38.7%)	
Yes	79 (63.2%)	33 (66.0%)	46 (61.3%)	
Complications	0.19 (0.40)	0.10 (0.30)	0.25 (0.44)	0.022 *
Ileostomy/colostomy ^##^:				0.965
No	96 (76.8%)	39 (78.0%)	57 (76.0%)	
Yes	29 (23.2%)	11 (22.0%)	18 (24.0%)	
Hospital stays (days)	7.38 (4.30)	6.78 (3.74)	7.78 (4.61)	0.185
Survival:				0.523
No	114 (91.2%)	47 (94.0%)	67 (89.3%)	
Yes	11 (8.80%)	3 (6.00%)	8 (10.7%)	

Data are expressed as mean ± standard deviations or as a percentage. Asterisk indicates significant difference between groups, according to the Welch two-sample test. The Chi-square test was used for variables expressed as a percentage (* *p* < 0.05). ^#^ Includes chemotherapy and/or radiotherapy. ^##^ Includes ileostomy and colostomy. Abbreviations: BMI: body mass index; BIA: bioelectrical impedance analysis; CRP: C reactive protein; FM: fat mass; HGS: hand-grip strength; PhA: phase angle; RF-CIR: circumference of quadriceps rectus femoris; RF-CSA: rectus femoris cross-sectional area; SAT: subcutaneous adipose fat of leg (L), superficial abdominal (S), and total abdominal (T); SMI: skeletal muscle index; SPA: standardized phase angle.

**Table 2:** Predictive value of phase angle on complications and sarcopenia in patients with colorectal cancer.

Variables	Complications		Sarcopenia	
	OR [95% CI]	OR [95% CI]	OR [95% CI]	OR [95% CI]
	Males	Females	Males	Females
Anthropometric				
BMI (kg/m^2^)	0.89 [0.78;1.02]	0.86 [0.70;1.06]	0.42 [0.24;0.74] *	0.63 [0.43;0.91] *
BIA				
PhA (°)	0.83 [0.47;1.45]	0.15 [0.03;0.81] *	0.42 [0.19;0.95] *	0.27 [0.07;1.07]
SPA (°)	0.93 [0.58;1.48]	0.53 [0.21;1.33]	0.48 [0.23;1.01]	0.55 [0.22;1.36]
Echography				
RF-CSA	0.72 [0.47;1.09]	0.22 [0.05;1.01]	0.51 [0.26;0.97] *	0.34 [0.09;1.28]
RF-CIR	0.83 [0.54;1.26]	0.69 [0.35;1.38]	0.74 [0.42;1.28]	0.86 [0.46;1.61]
L-SAT	1.30 [0.79;2.15]	0.82 [0.15;4.56]	0.29 [0.02;3.58]	0.03 [0.00;1.83]
S-SAT	0.22 [0.02;2.28]	0.51 [0.09;3.01]	0.10 [0.00;3.01]	0.20 [0.02;1.71]
T-SAT	0.46 [0.17;1.27]	0.82 [0.25;2.75]	0.58 [0.18;1.84]	0.04 [0.00;0.77] *
VAT	0.37 [0.06;2.36]	0.23 [0.01;7.34]	1.46 [0.19;11.3]	0.01 [0.00;13.3]
Functional				
RHG	0.95 [0.85;1.06]	0.88 [0.51;1.49]	1.01 [0.89;1.14]	0.93 [0.66;1.31]
LHG	0.87 * [0.76;1.00]	0.99 [0.67;1.45]	0.99 [0.87;1.11]	0.92 [0.68;1.25]
Inflammation				
C/A ratio	1.01 [0.98;1.04]	1.04 [0.99;1.09]	1.01 [0.97;1.06]	1.02 [0.98;1.06]

Data are expressed as odds ratio (OR) ± [95% confidence interval (CI) showing low value and high value]. Asterisk indicates significant value according to the logistic regression test (* *p* < 0.05). ‡ indicates Bonferroni correction, according to the variables included, =0.05/67 = 0.0007. Abbreviations: BMI: body mass index; C/A: C reactive protein/albumin ratio; RHG: right hand-grip strength LHG: left hand-grip strength; PhA: phase angle; RF-CIR: circumference of quadriceps rectus femoris; RF-CSA: rectus femoris cross-sectional area; SAT: subcutaneous adipose fat of leg (L), superficial abdominal (S) and total abdominal (T); SPA: standardized phase angle.

**Table 3:** Multiple regression analysis of phase angle as a predictor of complications and sarcopenia in colorectal cancer patients.

Variables	Complications		Sarcopenia	
	HR (SD)	HR (SD)	HR (SD)	HR (SD)
	Males	Females	Males	Females
BIA				
PhA (°)	−0.09 (0.34)	−2.52 (1.19) *	−2.29 (0.96) *	−5.26 (2.78) *
SPA (°)	−0.07 (0.23)	−1.08 (0.63)	−1.04 (0.50) *	−3.53 (1.88)
Echography				
RF-CSA	−0.27 (0.26)	−1.32 (0.81)	−0.14 (0.41)	−1.28 (1.10)
T-SAT	NA	NA	0.42 (1.03)	−2.46 (1.72)
Functional				
LHG	−0.45 (0.30)	252.2 (447)	NA	NA

Data are expressed as Hazard ratio (standard error). Data are adjusted for age and BMI. Asterisk indicates significant value according to the Cox regression analysis (* *p* < 0.05). ‡ indicates Bonferroni correction, according to the variables included, = 0.05/67 = 0.0007. Abbreviations: LHG: left hand-grip strength; PhA: phase angle; RF-CSA: rectus femoris cross-sectional area; SAT: subcutaneous adipose fat of superficial abdominal; SPA: standardized phase angle; NA: not available.

**Table 4:** Predictive value of phase angle on complications and sarcopenia in colorectal cancer patients.

	AUC	Cut-off	Sensitivity	Specificity	*p* Value
Complications in females					
PhA	0.893	4.85	80%	88.9%	0.001 *
Sarcopenia in males					
PhA	0.959	5.19	90.9%	92.3%	0.026 *
SPA	0.958	0.17	90.9%	92.3%	0.036 *

Receiver operating characteristic (ROC) for PhA and complications and sarcopenia in patients with colorectal cancer. Data are adjusted for age and BMI. Asterisk indicates significant value according to the ROC curve analysis (*p < 0.05). ‡ indicates Bonferroni correction, according to the variables included, =0.05/67 =0.0007. AUC, area under the ROC curve. Abbreviations: PhA: phase angle; SPA: standardized phase angle.

## Data Availability

Not applicable.

## Supplementary Materials

The following supporting information can be downloaded at: https://www.mdpi.com/article/10.3390/cancers15030847/s1: Supplementary Figure S1. Correlation plots are presented to show association between body measurements of (A) measurements methods and (B) body composition (X-axis) and body measurements (Y-axis) of all participants. A Pearson correlation test was conducted, and the asterisk indicates a significant correlation between variables according to this test (* *p* < 0.05). Colored squares without X indicate significant correlation between variables (* *p* < 0.05). Abbreviations: BMI: body mass index; BIA: bioelectrical impedance analysis; CRP: C reactive protein; C/A: CRP/albumin; FM: fat mass; HGS: hand-grip strength; PhA: phase angle; RF-CIR: circumference of quadriceps rectus femoris; RF-CSA: rectus femoris cross-sectional area; SAT: subcutaneous adipose fat of leg (L), superficial abdominal (S) and total abdominal (T); SMI: skeletal muscle index; SPA: standardized phase angle. Supplementary Figure S2. Correlation plots are presented to show association between body measurements (X-axis) and cancer outcomes (Y-axis) of (A) female, (B) male, and (C) all participants. A Pearson correlation test was conducted and the asterisk indicates significant correlation between variables according to this test (* *p* < 0.05). Abbreviations: BMI: body mass index; BIA: bioelectrical impedance analysis; CRP: C reactive protein; C/A: CRP/albumin; FM: fat mass; HGS: hand-grip strength; PhA: phase angle; RF-CIR: circumference of quadriceps rectus femoris; RF-CSA: rectus femoris cross-sectional area; SAT: subcutaneous adipose fat of leg (L), superficial abdominal (S) and total abdominal (T); SMI: skeletal muscle index; SPA: standardized phase angle. Supplementary Table S1: Summary descriptive table by groups of sex. Data are expressed as mean ± standard deviations or as a percentage. The asterisk indicates significant difference between groups, according to the Welch two-sample test (* *p* < 0.05). A chi-square test was used for variables expressed as a percentage (* *p* < 0.05). Abbreviations: BMI: body mass index; FM: fat mass; FMI: fat mass index; FFM: fat free Mass; FFMI: fat free mass index; MM: muscle mass; SMI: skeletal muscle index; SMM: skeletal muscle mass; ASMM: appendicular skeletal muscle mass; BCM: body cell mass; BCMI: body cell mass index; TBW: total body water; ECW: extracellular water; CRP: C reactive protein; Ratio C/A: PCR/albumin ratio.
