# Supplementary material for: Exploratory Assessment of Nutritional Evaluation Tools as Predictors of Complications and Sarcopenia in Patients with Colorectal Cancer
Source: Cancers (Basel). 2023 Jan 30;15(3):847. doi: 10.3390/cancers15030847 (PMC9913772; doi:10.3390/cancers15030847)
Supplement: Supplementary file 1 [file cancers-15-00847-s001.zip › Supplementary Table 1.pdf]

Supplementary Table 1: Summary descriptive table by groups of sex

	All	Female	Male	*p* value
	N:127	***N:51***	***N:76***	
Body measurements				
Hydration status	73.4 (0.32)	73.5 (0.33)	73.4 (0.30)	0.385
Nutrition status	893 (211)	712 (109)	1016 (171)	<0.001*
FFM (kg)	55.8 (10.8)	47.0 (5.87)	61.8 (9.23)	<0.001*
MM (kg)	26.1 (6.78)	20.1 (4.40)	30.2 (4.82)	<0.001*
FMI (kg)	7.61 (3.71)	9.41 (4.10)	6.39 (2.86)	<0.001*
FFMI	20.3 (2.76)	18.6 (2.09)	21.4 (2.60)	<0.001*
SMM (kg)	26.1 (6.78)	20.1 (4.40)	30.2 (4.82)	<0.001*
ASMM (kg)	20.8 (4.59)	17.2 (3.09)	23.2 (3.76)	<0.001*
BCM (kg)	29.6 (7.47)	23.5 (3.84)	33.6 (6.52)	<0.001*
BCMI (kg)	10.7 (2.12)	9.32 (1.39)	11.6 (2.03)	<0.001*
TBW (kg)	41.3 (8.33)	34.9 (5.21)	45.6 (7.19)	<0.001*
ECW (kg)	19.2 (3.79)	17.2 (3.19)	20.6 (3.55)	<0.001*
ECW/TBW	0.47 (0.05)	0.49 (0.04)	0.45 (0.04)	<0.001*
ECW/ICW	0.37 (0.09)	0.34 (0.10)	0.38 (0.09)	0.031*
ECW/BCM	0.67 (0.16)	0.74 (0.17)	0.63 (0.13)	<0.001*
Ratio C/A	8.24 (17.9)	5.85 (16.6)	9.98 (18.7)	0.291*

Data are expressed as mean ± standard deviations or percentage. Asterisk indicates significant difference between groups, according to Welch's two sample test (*$p<0.05$). Chi squared test was used for variables expressed as percentage (*$p<0.05$). **Abbreviations**: BMI: Body Mass Index; FM: Fat Mass; FMI: Fat Mass Index; FFM: Fat Free Mass; FFMI: Fat Free Mass Index; MM: Muscle Mass; SMI: Skeletal Muscle Index; SMM: Skeletal Muscle Mass; ASMM: Appendicular Skeletal Muscle Mass; BCM: Body Cell Mass; BCMI: Body Cell Mass Index; TBW: Total Body Water; ECW: Extracellular Water; CRP: C Reactive Protein; Ratio C/A: PCR/Albumin Ration.
