# Supplementary figures and images for: Exploratory Assessment of Nutritional Evaluation Tools as Predictors of Complications and Sarcopenia in Patients with Colorectal Cancer
Source: Cancers (Basel). 2023 Jan 30;15(3):847. doi: 10.3390/cancers15030847 (PMC9913772; doi:10.3390/cancers15030847)

A)

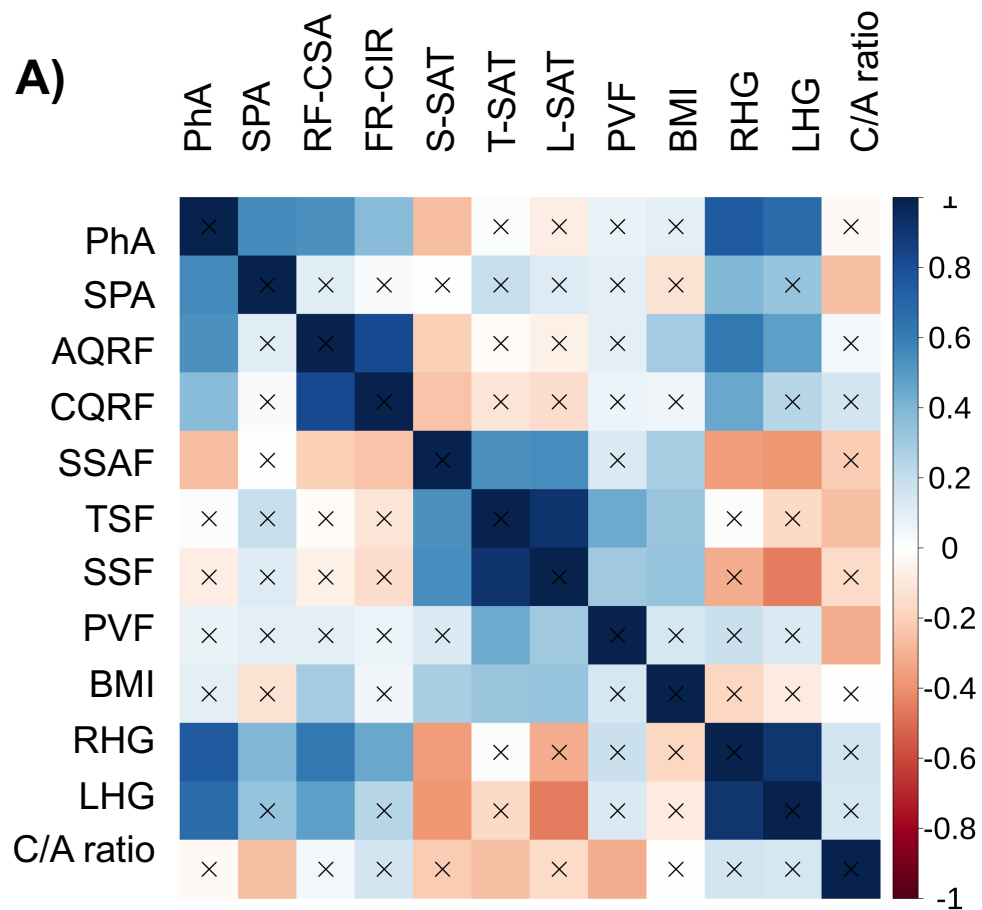

B)

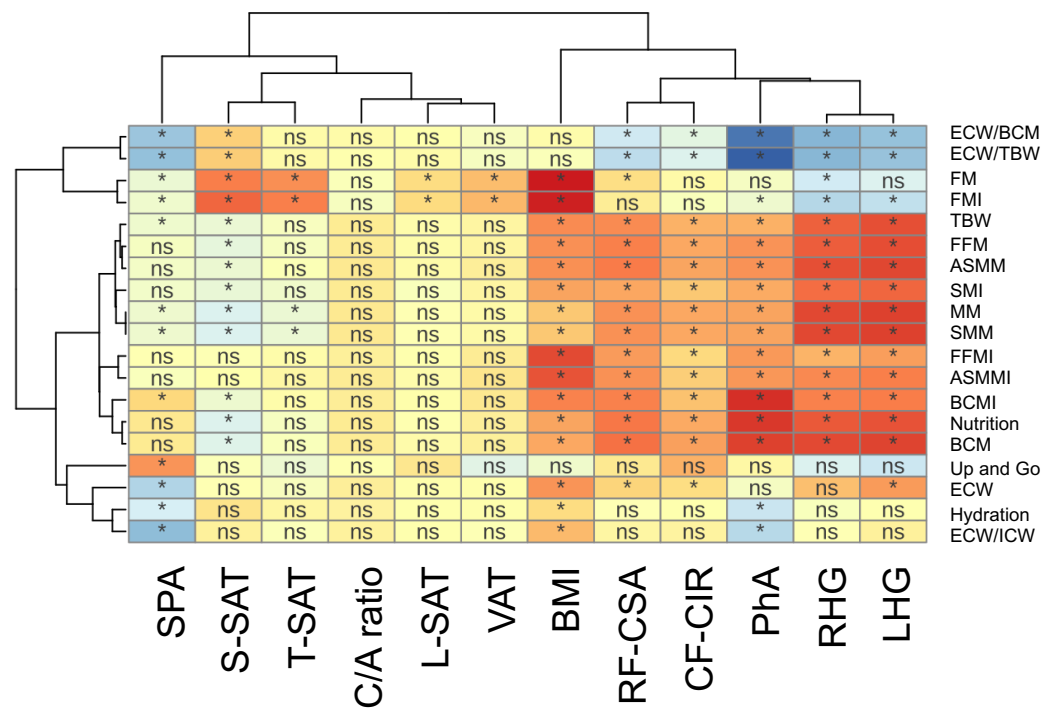

Supplement: Supplementary file 1 [file cancers-15-00847-s001.zip › Supplementary Figure 1.pdf]

A)

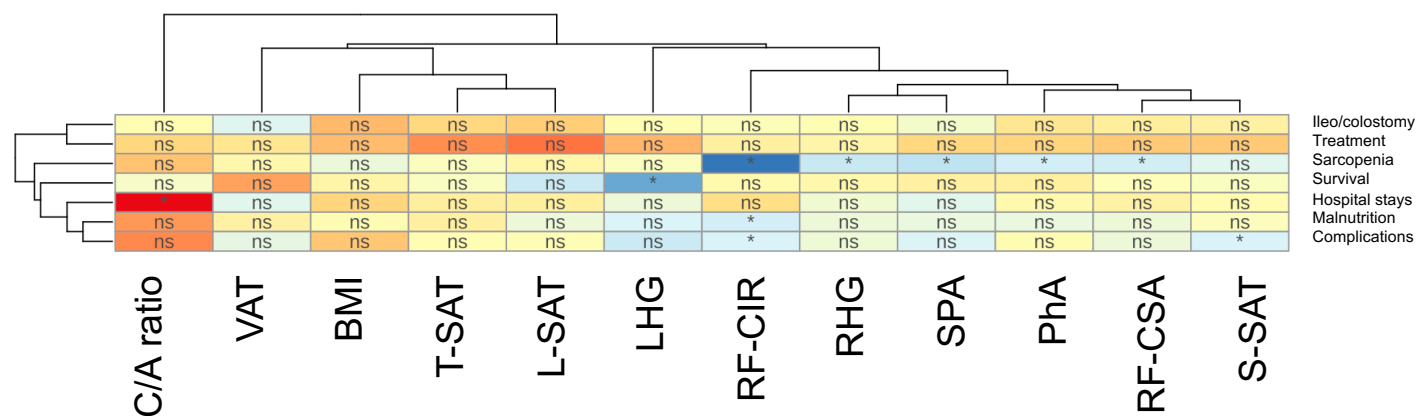

Correlation matrix

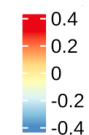

B)

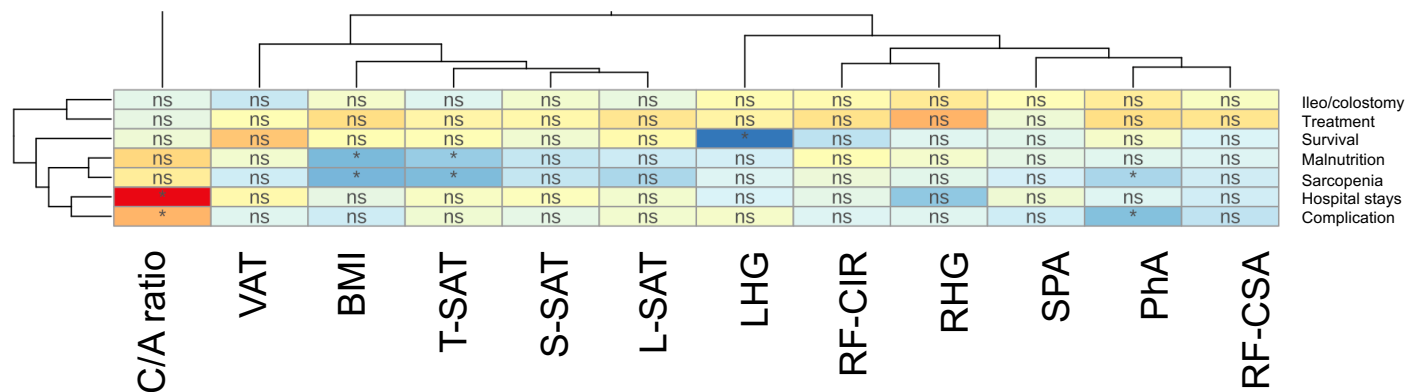

Correlation matrix

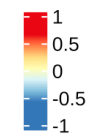

C)

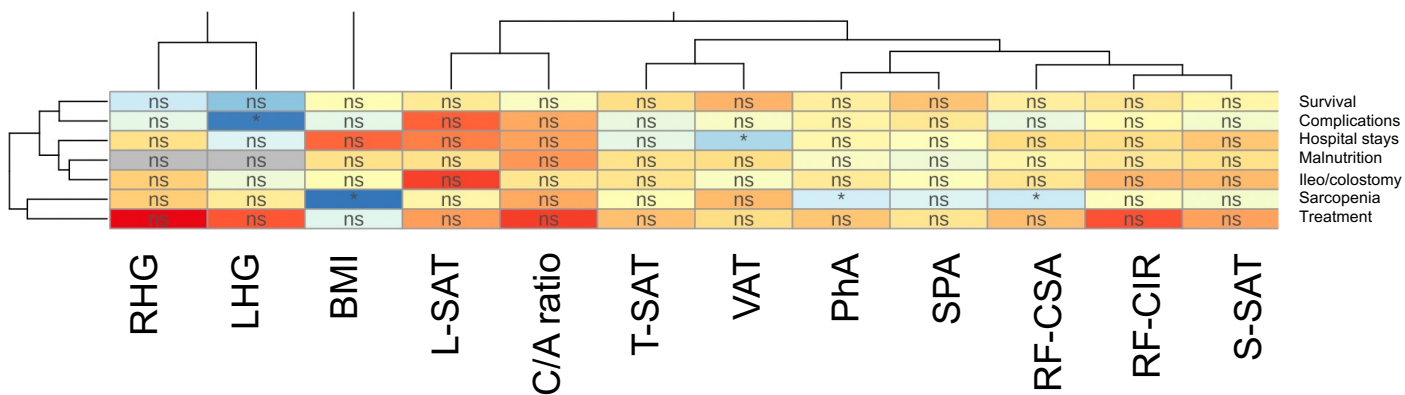

Correlation matrix

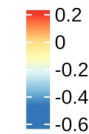

Supplement: Supplementary file 1 [file cancers-15-00847-s001.zip › Supplementary Figure 2.pdf]
